# 4-PBA inhibits endoplasmic reticulum stress to improve autophagic flux in the treatment of protamine/lipopolysaccharide-induced interstitial cystitis in rats

**DOI:** 10.1038/s41598-023-38584-x

**Published:** 2023-08-28

**Authors:** Li Jia, Zhu Jingzhen, Yang Xinliang, Sun Bishao, Luo Xin, Zheng Ji, Fang Zhenqiang

**Affiliations:** Department of Urology, Second Affiliated Hospital, Army Medical University, Chongqing, 40037 China

**Keywords:** Diseases, Pathogenesis

## Abstract

Interstitial cystitis (IC) has severe clinical symptoms with unclear mechanism. The continuous inflammatory response of the bladder is the basis of its pathogenesis. Endoplasmic reticulum stress (ERS) is involved in the regulation and development of various inflammatory diseases. And autophagy plays an important role in IC. In this study, we mainly focus on the therapeutic effect of endoplasmic reticulum stress and autophagy on protamine/lipopolysaccharide-induced interstitial cystitis. Female Sprague–Dawley rats were randomized into three experimental groups as follows: sham controls(N), IC alone, and IC+4-PBA.Rats in group IC received 10 mg/ml PS in the urinary bladder, followed by 2 mg/ml LPS instillation after 30 min, IC+4-PBA group SD rats received 4-PBA solution administered intragastrically once a day for 5 days. ERS biomarker (GRP78), autophagy-related proteins (LC3I/II, and Beclin1), autophagic flux biomarker (P62), inflammatory biomarkers (IL-6, TNF-a, NF-κB), apoptotic biomarkers (Caspase 3, Bax) were highest in the IC group compared to IC+4-PBA group and N group and the biomarkers expression in IC+4-PBA group were lower than in the IC group, anti-apoptotic biomarker (Bcl-2) was highest in the N group compared to the IC group and IC+4-PBA group and lower in the IC group than in the IC+4-PBA group, oxidative stress biomarkers (HO-1, NQO-1) were remarkably lower in the control group than in the IC and IC+4-PBA groups and notably lower in the IC group than in the IC+4-PBA group. The histological score and mast cell count demonstrated most severe in the IC group than those in the IC+4-PBA group. TUNEL assay examined the level of apoptosis in IC group was higher than in the IC+4-PBA group. The bladder micturition function was significantly improved with 4-PBA treatment. 4-PBA inhibits ERS to recover autophagic flux, and then to suppress the bladder oxidative stress, the inflammatory reaction and apoptosis, finally improve the bladder urinary function in Protamine/Lipopolysaccharide (PS/LPS) induced IC.

IC presents a variety of clinical phenotypes and different potential etiologies, such as urgency of urination, frequency of urination, pain in suprapubic area or pelvic cavity after bladder filling, and relief after urination^1^. It is a debilitating, incurable, and costly pain condition affecting approximately 3–8 million individuals in the United States^2^.Because the etiology of the syndrome and the diverse sites and degrees of symptoms were not clear, there have been only a few effective treatments reported so far. So exploring the pathogenesis of IC is of great significance in providing relevant ideas for the treatment of IC.

The endoplasmic reticulum (ER) is specializing in maintaining protein homeostasis. It is mainly recognized as a protein-folding factory which conducts the biosynthesis, folding, assembly and modification of numerous soluble proteins and membrane proteins^3^. ER stress (ERS) means to perturb protein processing, which leads to accumulation of misfolded proteins in the ER. Long term and severe ERS can induce cell apoptosis or death. The protein named glucose-regulated protein 78(GRP78) is an important biomarker of the ERS^4^.Studies have found that ERS plays an important role in the inflammatory response. ERS participates in the occurrence and development of various inflammatory diseases^5,6^. IC is viewed as an inflammatory disease, and ERS may be involved in regulating the occurrence and development of IC.

Autophagy is a dynamic process of degradation of long-lived proteins, cellular macromolecules, and intracellular organelles by the lysosomes^7^.The complete process of autophagy including the delivery of cargoto lysosomes (via fusion of the latter with autophagosomes or amphisomes) and its subsequent break down and recycling is called Autophagic flux^8^.The degradation of multifunctional protein p62 mainly depends on autophagy, so inhibition of autophagy will lead to the increase of p62^9^. Autophagic flux is impaired by ERS in several disease, such as non-alcoholic fatty liver disease (NAFLD)^10^ and intestinal inflammation^11^.Based on previous findings, we aim to research the mechanism of ERS and autophagic flux in the occurrence and development of PS/LPS-induced IC in a rat model.

## Materials and methods

### Ethics

This study was approved by the Research Council and Animal Care and Use Committee of the Army Medical University, China. All methods were performed in accordance with the relevant guidelines and regulations. A total of 55 female Sprague–Dawley (SD) rats weighing 200–230 g was used in this study and kept under standard laboratory conditions. The study is reported in accordance with ARRIVE guidelines.

### Study design

Ten female Sprague–Dawley (SD) rats were randomized into two experimental groups (5 rats each): sham controls (N) and IC alone. Five female SD rats from each group were tested for western blot detection of autophagy-related proteins (LC3I/II, and Beclin1) andautophagic flux biomarker (P62). Fourty-five female Sprague–Dawley (SD) rats were randomized into three experimental groups (5 rats each): Sham controls (N), IC alone and IC+4-PBA(ERS inhibitor, 4-Phenylbutyric acid)^12^. Five female SD rats from each group were tested for western blot detection of ERS biomarker (GRP78), autophagy-related proteins (LC3I/II, and Beclin1), autophagic flux biomarker (P62), inflammatory biomarkers (IL-6, TNF-a, NF-κB), apoptotic biomarkers (Caspase 3, Bax), anti-apoptotic biomarkers (Bcl-2) and oxidative stress biomarkers (HO-1, NQO-1). Each group of 5 female SD rats was sacrificed for histological analysis: HE and toluidine blue staining, and for TUNEL staining. The remaining 5 female SD rats of each group were prepared for cystometry assessments.

### Animal model establishment

The rats in group N were continuously perfused with normal saline in bladders. In IC group, bladders were perfused with 10 mg/ml protamine for 30 min, then washed with normal saline for three times, then perfused with 2 mg/ml LPS for 45 min, and finally washed for three times. The IC model was established 5 days later. The IC+4-PBA group was treated with 4-PBA of 500 mg/kg/d by gavage for 5 consecutive days when the rat model was established^13,14^. The rats in each group were anesthetized by intraperitoneal injection of 10% chloral hydrate and then perfused through urethra and bladder.

### Quantitative analysis of ERS, autophagy, autophagic flux, inflammatory, apoptotic, anti-apoptotic and oxidative stress biomarkers in bladders using western blot

The bladder tissue (n = 5) in each group was used for western blotting. We used whole protein extraction kit for extracting total protein from bladder tissue. Western blot was used to detect the expression of GRP78(Abcam, ab21685), LC3I/II(Cell Signaling Technology, #12741),Beclin1(Cell Signaling Technology, #3495), P62(Abcam, ab109012), IL-6(GeneTex, GTX110527), TNF-a(Abcam, ab205587), NF-κB(Cell Signaling Technology, #8242), Caspase 3(GeneTex, GTX110543), Bax(GeneTex, GTX109683), Bcl-2(GeneTex, GTX100064), HO-1(Abcam, ab68477) ,and NQO-1(Abcam, ab80588). The specific method was reported in the former research^15^. All the blots were cut before hybridisation with antibodies. The protein band images were collected. And we used ChemiDoc XRS+Image System (Bio-Rad Laboratories, Hercules, CA, USA) to analyse the relative optical density (R.O.D).

### Histological evaluation

The bladder wall of rats (n = 5) of each group was sectioned (5 μm per slice) for histological analysis. HE and toluidine blue staining were applied. Subsequently, the histological score and mast cell counts were determined by an investigator in a blinded fashion. Histological slides were graded by a score of 0–5 as previous reported^16^. The quantification of mastocytes in the lamina propria and muscle layer was estimated at 200× magnification in five random sections from each group.

### TUNEL staining

Detection of apoptosis by a TUNEL staining assay kit purchased from Roche Applied Science on paraffin sections of the bladder of three groups. We carried out the quantitation of the positive cells number at a magnification of 200× in five randomly chosen fields of view on each slide. The apoptosis index was calculated using the ratio of the apoptotic cells to total cells.

### Bladder cystometry

Cystometry was performed in N, IC andIC+4-PBAgroups for 5 SD rats of each, similar to the method described previously^17^.First, we place the PE-50 catheter into the bladder, then body temperature (37–38 °C) saline was infused at a rate of 0.1 ml/min using a syringe pump. Intravesical pressure was recorded continuously, after a stabilizing time of approximately 30 min, urodynamic parameters including basal pressure (BP), maximum pressure (MP), and micturition frequency (MF)were evaluated.

### Statistical analysis

SPSS16.0 (SPSS) was used for all analyses by an investigator blinded to the treatment groups. All outcomes were analyzed using one-way ANOVA, followed by Fisher’s test to assess differences among treatment groups. A probability value of < 0.05 was considered significant.

## Results

### Confirmation of the differences of autophagy and autophagic flux with western blot in N and IC groups

**Figure 1:**
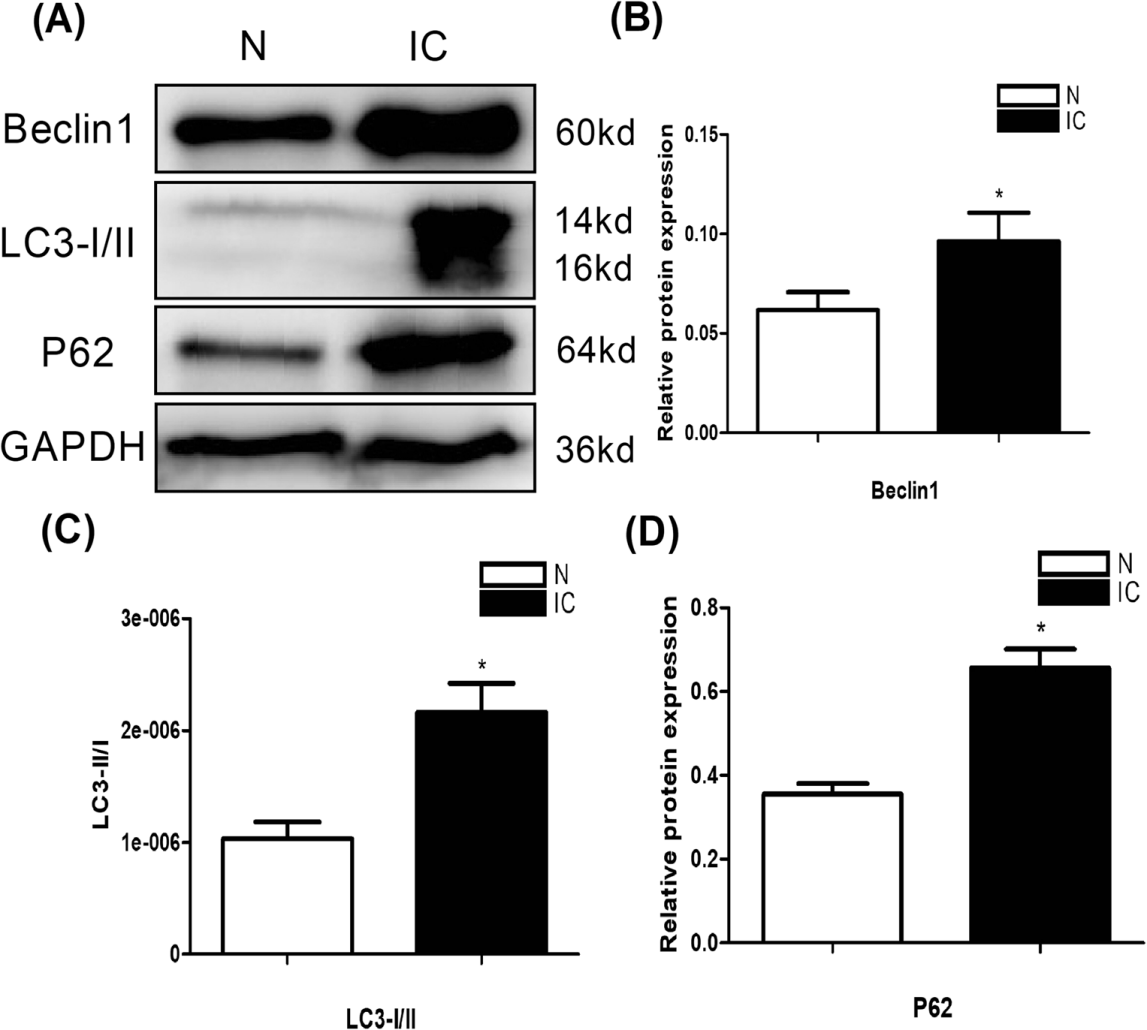
Expression of autophagy and autophagic flux factors in the urinary bladder at 5 days after IC induction (n = 5). (**A**) Expression of the autophagy and autophagic flux biomarkers LC3I/II, Beclin1, and P62 by western blot in the bladder tissue were higher in the IC group compared to normal control groups. All the blots were cut before hybridisation with antibodies. (**B**) A statistical chart of the relative optical density of Beclin1 /GAPDH in each group (n = 5). (**C**) A statistical chart of the relative optical density of LC3II / LC3I /GAPDH in each group (n = 5). (**D**) A statistical chart of relative optical density of P62/GAPDH in each group (n = 5). * indicates a significant difference compared to the control group value (*P* < 0.05).

The expression of the autophagy biomarkers LC3I/II, and Beclin1 by western blot in the bladder tissue is higher in the IC group compared to control group. Also, the P62 expression, which is one indicator of autophagic flux, is higher in the IC group compared to control group (Fig. 1A–D).The results suggest that autophagy is enhanced in IC, but autophagic flux is inhibited.

### Evaluation of ERS, autophagy and autophagic flux factors after 4-PBA treatment

**Figure 2:**
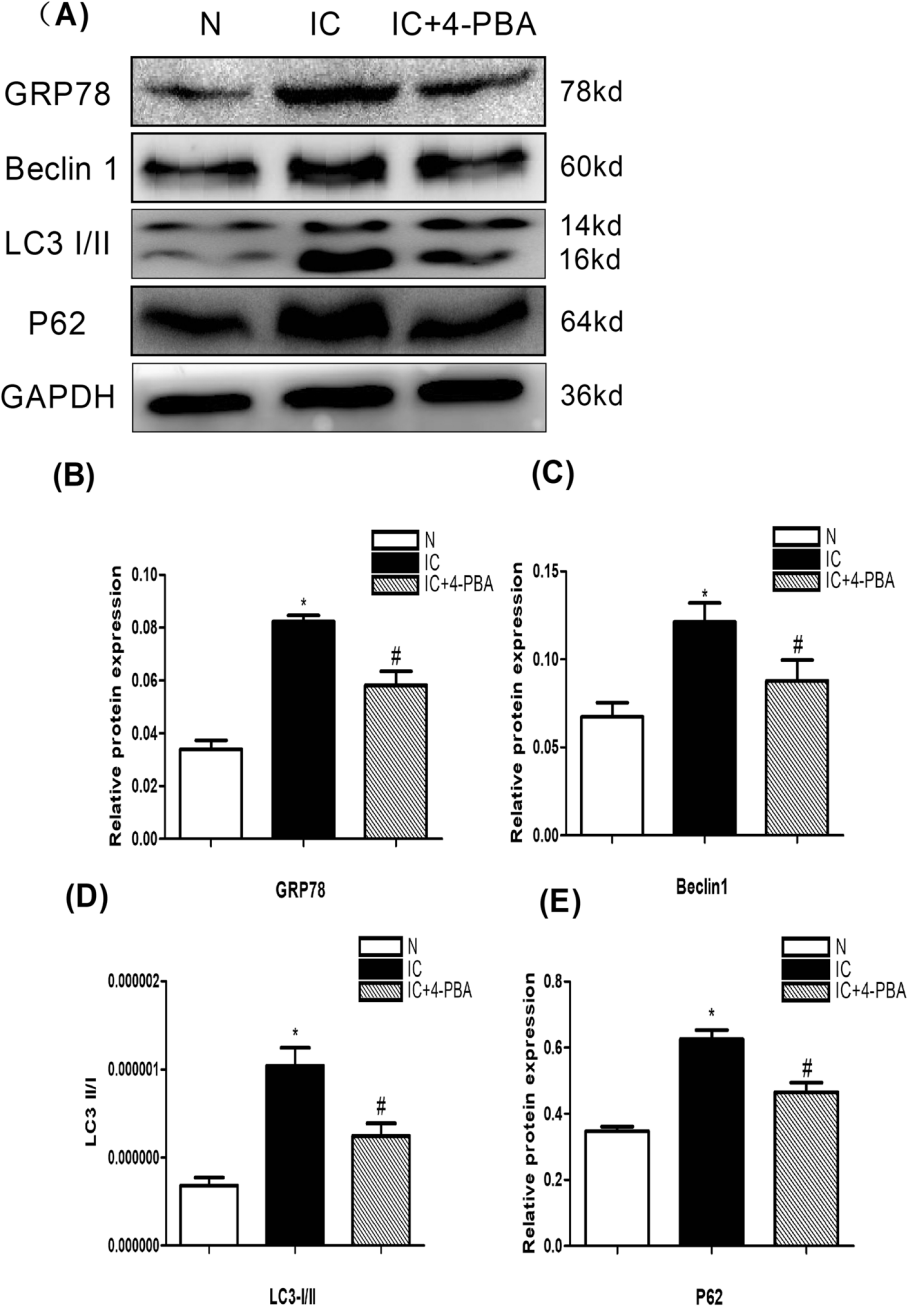
Analysis of protein expression of ERS biomarker (GRP78), autophagy biomarkers (LC3I/II, Beclin1) and autophagic flux biomarker (P62) in the urinary bladder at 5 days after IC induction (n = 5). (**A**) Expression of the a ERS biomarker (GRP78), autophagy biomarkers (LC3I/II, Beclin1) and autophagic flux biomarker (P62) by western blot in the bladder tissue were highest in the IC group compared to IC+4-PBA group and normal control groups; the biomarkers expression in IC+4-PBA group was lower than in the IC group. All the blots were cut before hybridisation with antibodies. (**B**) A statistical chart of the relative optical density of GRP78/GAPDH in each group (n = 5). (**C**) A statistical chart of the relative optical density of Beclin1/GAPDH in each group (n = 5). (**D**) A statistical chart of the relative optical density of LC3II / LC3I /GAPDH in each group (n = 5). (**E**) A statistical chart of the relative optical density of P62/GAPDH in each group (n = 5). * indicates a significant difference compared to the control group value (*P* < 0.05). # indicates a significant difference compared to the IC group value (*P* < 0.05).

The ERS biomarker (GRP78), autophagy biomarkers (LC3I/II, Beclin1) and autophagic flux biomarker (P62) expression using western blot are highest in the IC group. After 4-PBA treatment, the expressions of all former biomarkers reduce in IC + 4-PBA group (Fig. 2A–E). These results reveal that ERS is enhanced in IC, and autophagic flux is improved after inhibition of ERS.

### Evaluation of inflammation-related factors and oxidative stress-related factors

**Figure 3:**
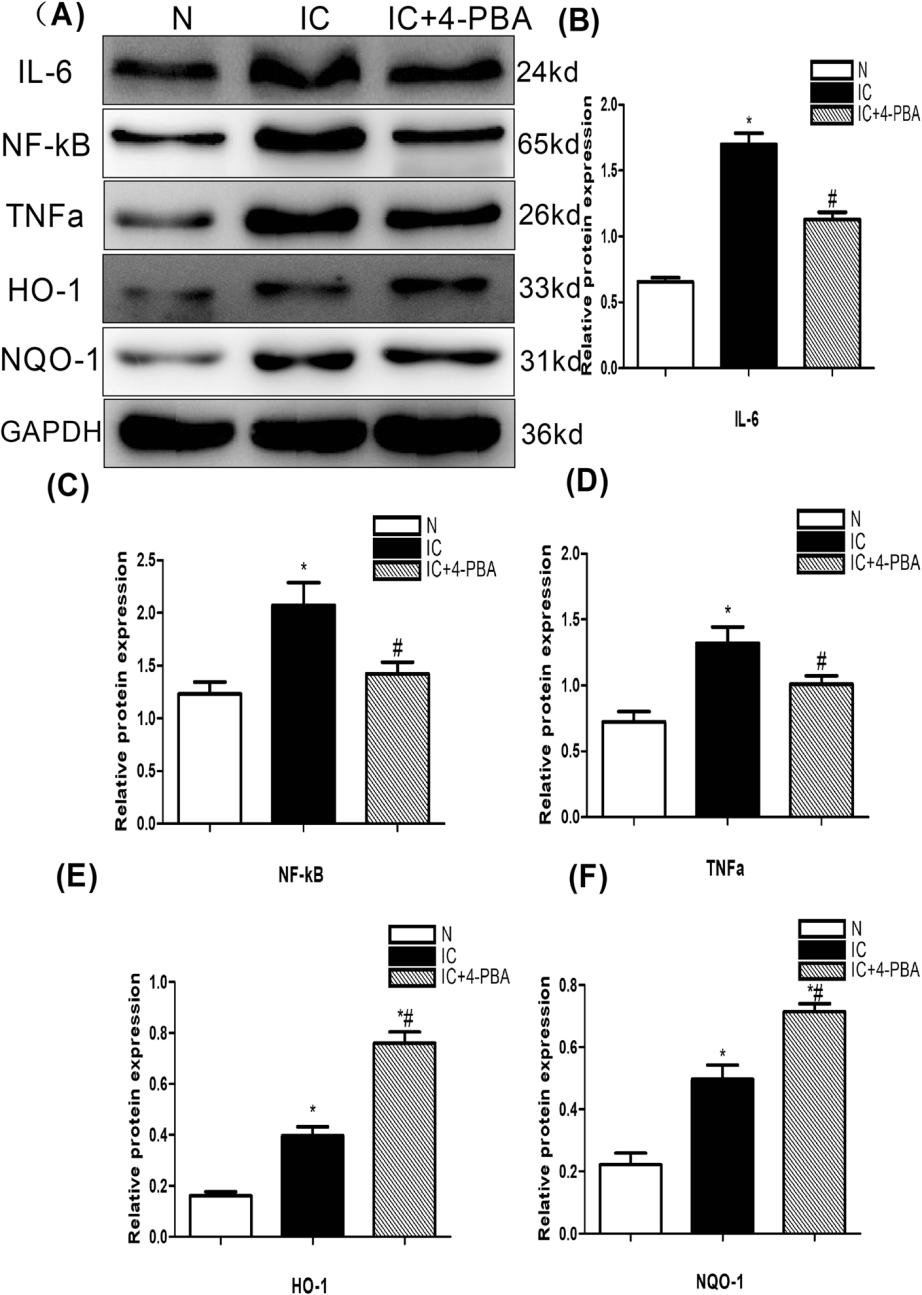
Expression of inflammatory-related factors and oxidative stress-related factors in the urinary bladder at 5 days after IC induction (n = 5). (**A**) Expression of the inflammation-related factors IL-6, TNF-a, NF-κB by western blot in the bladder tissue was highest in the IC group compared to IC+4-PBA group and normal control groups; the biomarkers expression in IC+4-PBA group was lower than in the IC group. The oxidative stress-related factors HO-1 and NQO-1 by western blot was remarkably lower in the control group than in the IC and IC+4-PBA groups and notably lower in the IC group than in the IC+4-PBA group. All the blots were cut before hybridisation with antibodies. (**B**) A statistical chart of the relative optical density of IL-6/GAPDH in each group (n = 5). (**C**) A statistical chart of the relative optical density of NF-κB /GAPDH in each group (n = 5). (**D**) A statistical chart of relative optical density of TNF-a /GAPDH in each group (n = 5). (**E**) A statistical chart of relative optical density of HO-1/GAPDH in each group (n = 5). (**F**) A statistical chart of relative optical density of NQO-1/GAPDH in each group (n = 5). * indicates a significant difference compared to the control group value (*P* < 0.05). # indicates a significant difference compared to the IC group value (*P* < 0.05).

The protein expression of three inflammation-related factors IL-6,TNF-a and NF-κB by western blot in the control group is remarkably lower than in the IC group and the figure inIC+4-PBA group is lower than inthe IC group. The two anti-oxidative indicators oxygenase (HO)-1 and NAD(P)H quinone oxidoreductase (NQO)-1 present in IC group is increased than in the control group, after 4-PBA treatment, the HO-1 and NQO-1expressionwere significantly enhanced compared to the IC group (Fig. 3A–F).These results reveal that the level of inflammatory reaction and oxidative stress are inhibited inhibition of ERS.

### The quantitative assessment of the histological score and mast cell count

**Figure 4:**
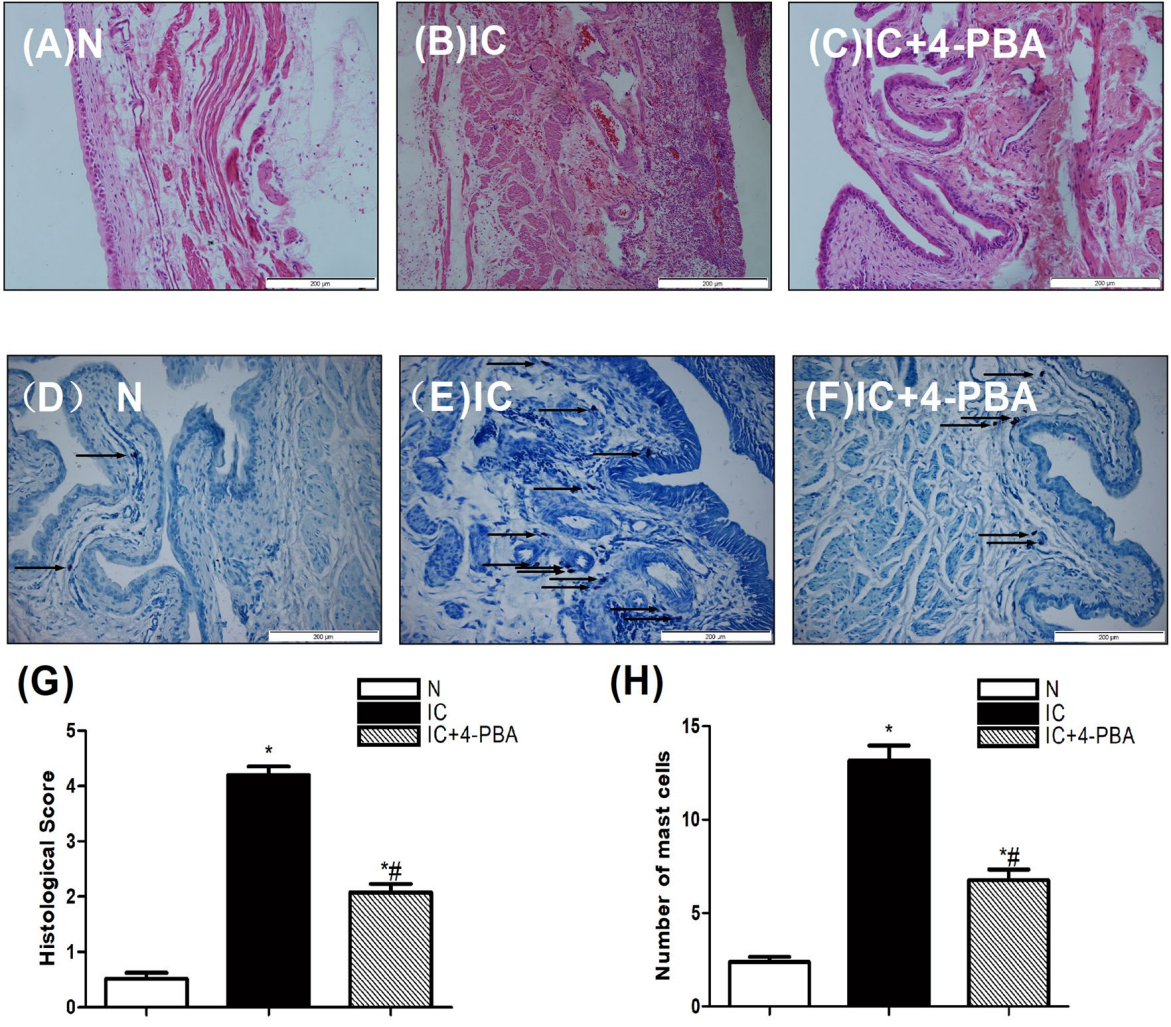
The quantitative assessment of the histological score and mast cell count in the urinary bladder at 5 days after IC induction (n = 5). (**A**–**C**) Photomicrograph images of H&E staining in rat bladder samples (scale bars are 200 μm). (**D**–**F**) Representative photomicrograph images of rat bladder samples stained with toluidine blue (arrows) demonstrate mast cells (scale bars are 200 μm). (**G**) The statistical chart demonstrates the inflammation grading (n = 5). (**H**) A statistical chart reveals the number of mast cells in the bladder of rats (n = 5). * Indicates a significant difference compared to the control group value (*P* < 0.05). # indicates a significant difference compared to the IC group value (*P* < 0.05).

There are massive ulcers, obvious edema, hemorrhage and increased inflammatory cell infiltration (particularly mast cells) in the sub-mucosal and muscular layer of the bladder tissue from the IC group rats compared with the control group. In the IC+4-PBA group, this situation is significantly improved (Fig. 4A–C). The quantitative of the mast cell count and histological score are reduced in the IC group than those in the IC+4-PBA group (Fig. 4D–H).This results demonstrate that the inflammatory response was the most severe in the IC group, it is significantly alleviated after inhibition of ERS.

### Protein expression of apoptotic mediators

**Figure 5:**
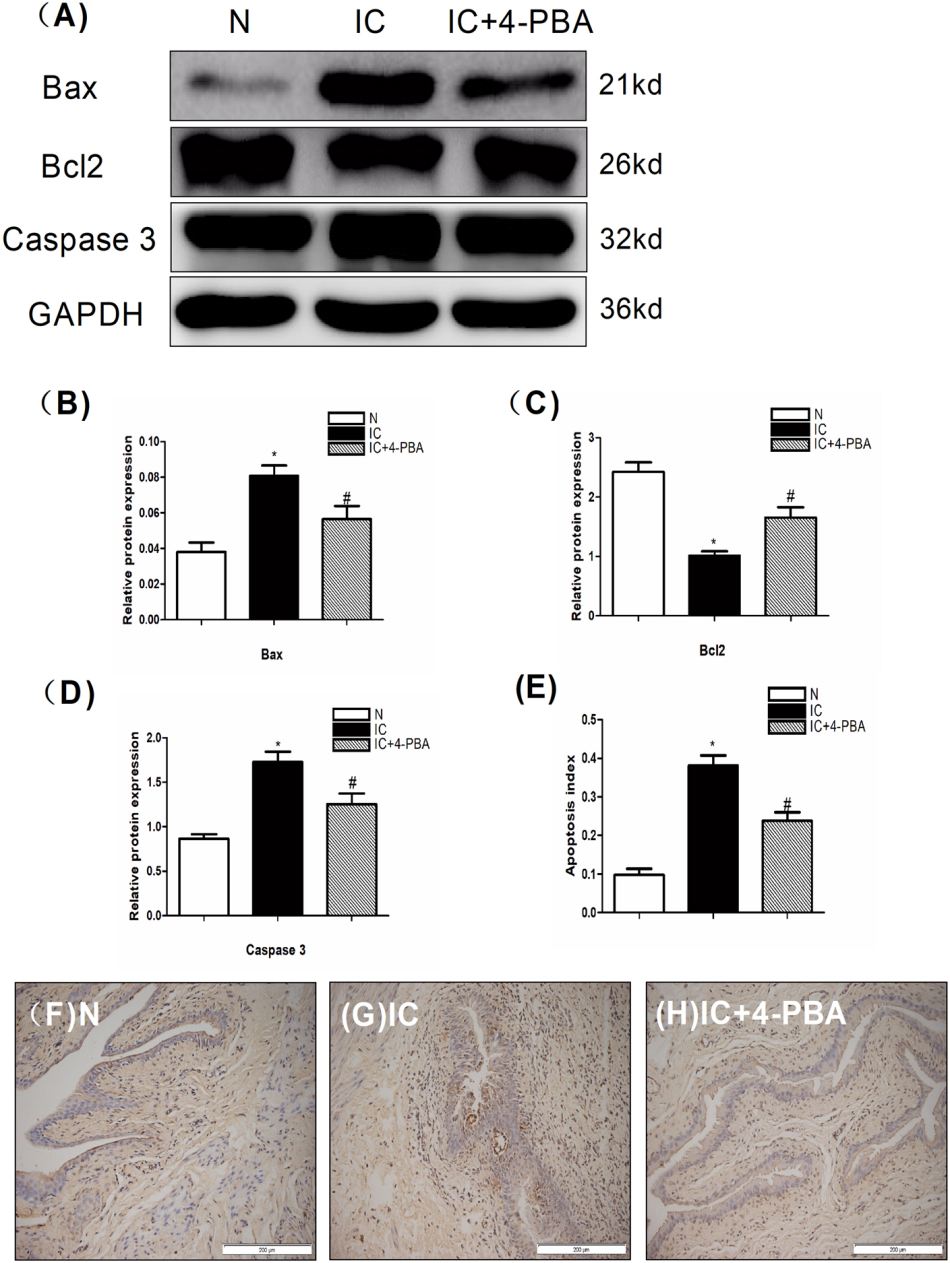
Analysis of protein expression of the apoptosis biomarkers Bax and Caspase 3 and the anti-apoptosis indicator BCL-2 and a TUNEL assay in the urinary bladder at 5 days after IC induction (n = 5). (**A**) Expression of the apoptosis biomarkers Caspase 3 and Bax by western blot in the bladder tissue was highest in the IC group compared to the IC+4-PBA group and normal control group; the biomarker expression in the IC+4-PBA group was lower than in the IC group. Expression of anti-apoptosis indicator BCL-2 was highest in the normal control group compared to the IC group and IC+4-PBA group, lower in the IC group than in the IC+4-PBA group. All the blots were cut before hybridisation with antibodies. (**B**) A statistical chart of the relative optical density of Bax/GAPDH in each group (n = 5). (**C**) A statistical chart of the relative optical density of BCL-2/GAPDH in each group (n = 5). (**D**) A statistical chart of the relative optical density of Caspase 3/GAPDH in each group (n = 5). (**E**) A statistical chart reveals the index of apoptotic nuclei in all groups of bladder tissue (n = 5). (**F**–**H**) The TUNEL assay indicated that in sections from the IC group rats, the most apoptotic nuclei were observed, whereas fewer were present in the IC+4-PBA group and more were present than in the normal control group. * indicates a significant difference compared to the control group value (*P* < 0.05). # indicates a significant difference compared to the IC group value (*P* < 0.05).

Two indicators of apoptosis, Caspase 3 and Bax, are significantly higher in the IC group than in the N group by western blot. And the IC+4-PBA group is markedly lower than the IC group. Furthermore, the index of anti-apoptosis (Bcl-2 protein expression) was remarkably lower in the IC group, this indicator increases in the IC+4-PBA group and is lower than in the N group (Fig. 5A–D). We also use TUNEL staining to examine the apoptosis levels in each group. The most quantity of apoptotic nuclei are observed in the bladder sections of IC group rats, and fewer were present in the IC+4-PBAgroup and more present in the N group (Fig. 5E–H). These results reveal that inhibiting ERS could alleviate apoptotic effects of IC.

### Changes in urodynamic parameters

**Figure 6:**
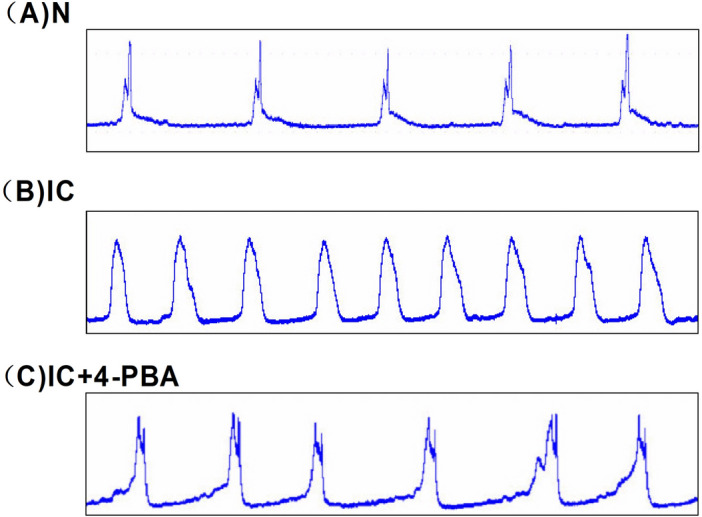
Cystometry variables of the sham control (N), IC, and IC+4-PBA groups.

Compared to the control group, basal pressure and micturition frequency per hour are significantly increased in IC group compare to the N group, they are decreased in the IC+4-PBA group than the IC group. No statistical significance was found in the maximum pressure compared between the three groups. These results indicate a recovered micturition function of IC after ERS impeded (Table 1 and Fig. 6).

**Table 1:** Cystometric parameters changes in the sham control (N), IC, and IC+4-PBA groups.

Urodynamic Parameters	N	IC	IC + 4-PBA
Basal pressure (cm H2O)	2.86 + 0.66	24.76 + 3.3*	15.61 + 1.62#
Maximum pressure (cm H2O)	72.15 + 5.21	27.8 + 4.01	41.09 + 2.31
Micturition frequency (No/h)	10.17 + 1.46	24.31 + 3.58*	14.92 + 1.81#

Data presented as means ± SD (n = 5).

*Indicates a significant difference compared to the control group value (*P* < 0.05).

^#^Indicates a significant difference compared to the IC group value (*P* < 0.05).

## Discussion

In this study, the expression of the autophagy biomarkersLC3I/II and Beclin1 by western blot in the bladder tissue is higher in the IC group. Also, the autophagic flux indicator P62 expression is higher in the IC group. So the autophagy was enhanced in IC, but autophagic flux was blocked. Autophagic flux refers to the complete process of autophagy including the delivery of cargoto lysosomes and its subsequent breakdown and recycling^8^. Autophagic flux blocking means that the function of autophagy scavenging waste was reduced. Research showed autophagy agonist RAPA could significantly decrease inflammation and improve bladder function of IC, and it may be used as a potential treatment for IC^18^. Based on our research, this result may be caused by the recovery of autophagic flux.

Blocked autophagic flux participates in the occurrence and development of many diseases. In pregnancy, the blockade of autophagic flux takes part of disrupting homeostasis in trophoblasts^19^. In glioma, the autophagic flux is impaired^20^. The autophagic flux damaged in former diseases are related to ERS. ERS can reduce the abnormal aggregation of proteins in cells by activating the unfolded protein response (UPR), thus playing a role in cell protection. However, long-term and severe ER stress can cause cell apoptosis or death. In our study, the expression of ER stress markerGRP78was enhanced in IC. This indicated that the ERS was enhanced in IC. Then we use ERS inhibitor, 4-Phenylbutyric acid, to treat the IC rats. We found autophagic flux biomarker (P62) reduced. This result indicated that inhibiting ERS could improve the autophagic flux in IC, subsequently the microenvironment of bladder became better, and then autophagy biomarkersLC3I/II and Beclin1 reduced.

ERS inhibiting autophagic flux is the key pathogenic factor of many diseases. HCV-induced ER stress correlates with autophagic flux impairment. Decrease of ER stress improved autophagic flux impairment is considered to be a promising therapeutic strategy for HCV-related chronic liver diseases^21^.Autophagy plays a critical role in the development of non-alcoholic fatty liver and steatohepatitis. ERS inhibiting autophagic flux contributes to high-fat, high-fructose, and high-cholesterol diet-induced liver injury and inflammatory response^22^.In our study, inhibition of ERS improved autophagic flux, the expression of inflammation-related factors (IL-6, TNF-a, NF-κB) and apoptosis indicator(Caspase 3 and Bax) were decreased. Anti-oxidative indicators(HO-1, NQO-1) and anti-apoptosis indicatorBcl-2 were remarkably increased. Histological score, mast cell and apoptotic nuclei count were declined. Finally, the micturition function of IC was recovered after ERS impeded. The application of 4-PBA inhibiting ER stress to restore autophagic flux could improve the bladder urination function of IC. But the specific mechanism is still unclear.

Disruption of ER homeostasis and the accumulation of unfolded or misfolded proteins elicit ERS, subsequently activate downstream signaling pathways: Protein Kinase R-like ER Kinase (PERK), Inositol Requiring Enzyme1α (IRE1α), and Activating Transcription Factor 6 (ATF6)^23,24^. Numerous studies have demonstrated that ERS affects autophagic flux through IRE1 signaling pathway. In the study of insulin resistance, activating ERS could inhibit autophagy flux through IRE1 signal pathway, leading to insulin resistance^25^.ERS inhibits autophagy flux by IRE1signaling pathway, leading to the accumulation of mutant huntingtin protein aggregates and neurotoxicity in Huntingtin^26^.IRE1 interferes with autophagic flux mainly through the following ways: 1. ERS makes autophagosomes and lysosomes blend through Rab7^27^. 2. ERS inhibits autophagic flux by inhibiting lysosomal function through IRE1 pathway^28^. 3. Since the double membrane structure of autophagosome is derived from the endoplasmic reticulum/Golgi theory, IRE1 regulates the production of endoplasmic reticulum/Golgi, and the ERS IER1 pathway regulates the formation of double membrane structure of autophagosome^29^.Therefore, ERS may block autophagic flux by IRE1 pathway to affect bladder function of IC.

In summary, the results of this study revealed that 4-PBA inhibiting ERS recover autophagic flux. The ability of autophagy removing waste was enhanced, which improved the microenvironment of bladder, ultimately significantly improved the bladder micturition function. It provides a new perspective and supplement for the pathogenesis and treatment of IC (Supplementary file).

### Supplementary Information

Supplementary Information.

## Data Availability

The datasets used and/or analysed during the current study available from the corresponding author on reasonable request.
