# Supplementary material for: 4-PBA inhibits endoplasmic reticulum stress to improve autophagic flux in the treatment of protamine/lipopolysaccharide-induced interstitial cystitis in rats
Source: Sci Rep. 2023 Aug 28;13:14057. doi: 10.1038/s41598-023-38584-x (PMC10462651; doi:10.1038/s41598-023-38584-x)
Supplement: Supplementary file 1 — Supplementary Information. [file 41598_2023_38584_MOESM1_ESM.docx]

Fig.1.

(1)

Beclin 1

N1 IC1 N2 IC2 N3 IC3


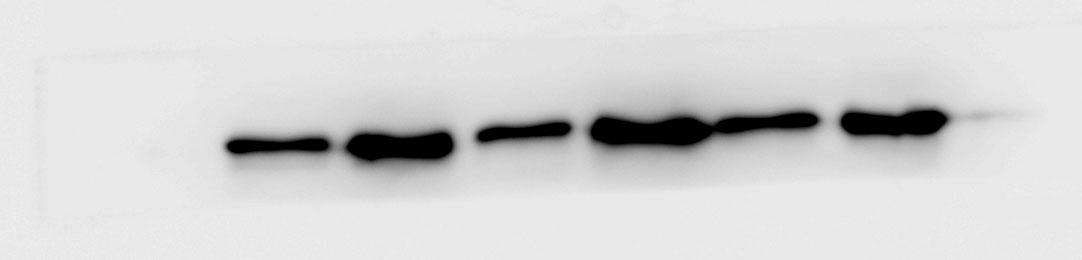


LC3I/II

N1 IC1 N2 IC2 N3 IC3


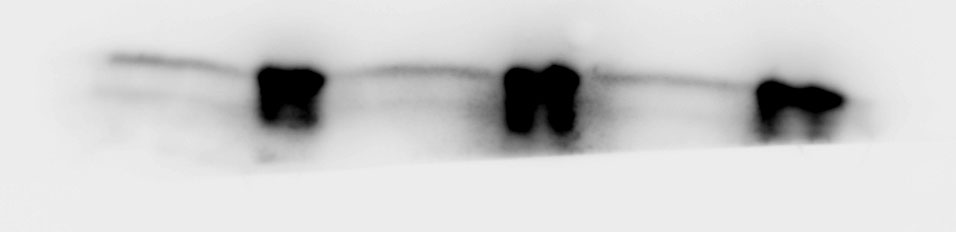


P62

N1 IC1 N2 IC2 N3 IC3


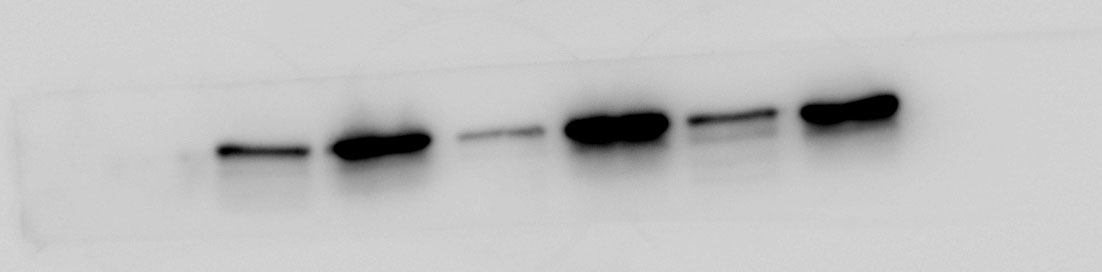


GAPDH

N1 IC1 N2 IC2 N3 IC3





(2)

N IC


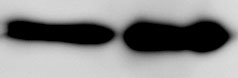
 Beclin1


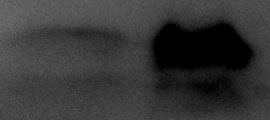
 LC3I/II


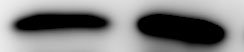
 P62


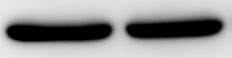
 GAPDH

Fig.2

GRP78

N IC IC+4-PBA


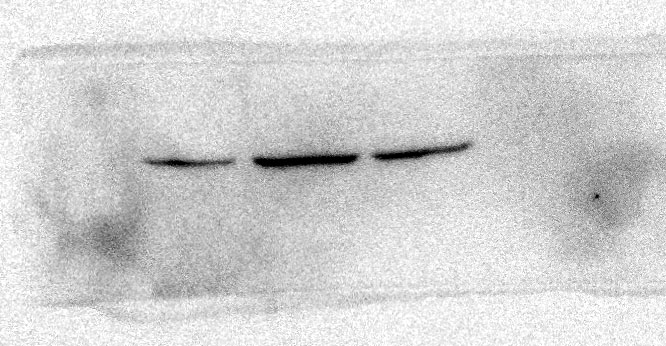


Beclin 1

N IC IC+4-PBA


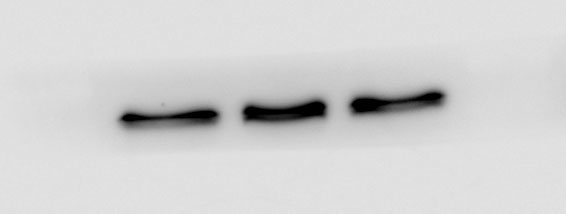


LC3I/II

N IC IC+4-PBA


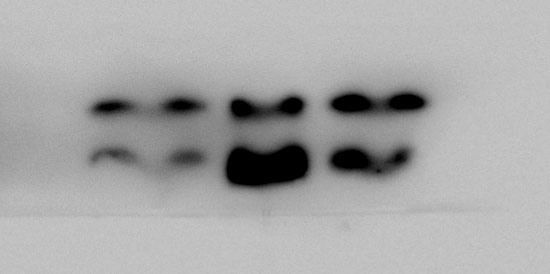


P62

N IC IC+4-PBA


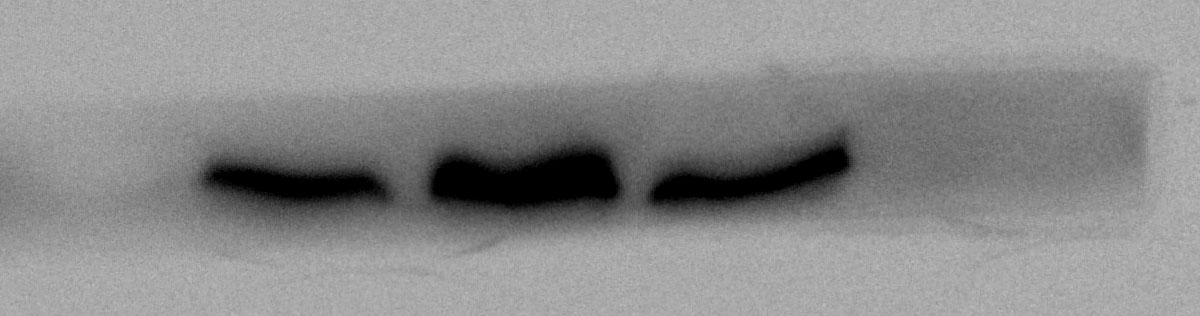


GAPDH

N IC IC+4-PBA

(2).

N IC IC+4-PBA


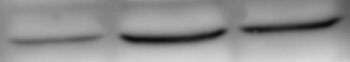
 GRP78


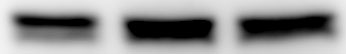
 Beclin1


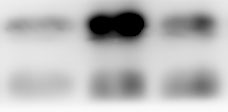
 LC3I/II


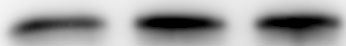
 P62

GAPDH

(3).

N IC IC+4-PBA


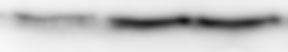
 GRP78


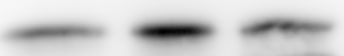
 Beclin1


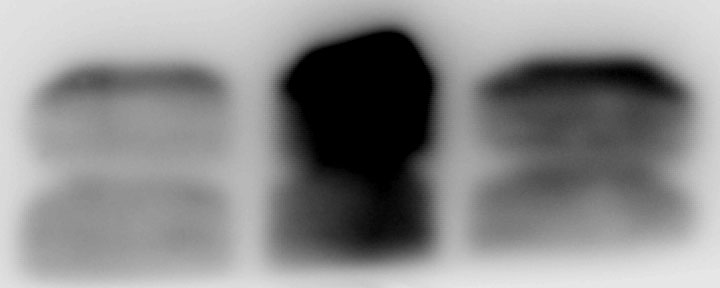
 LC3I/II


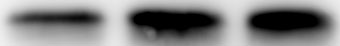
 P62


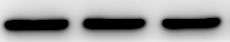
 GAPDH

(4).

N IC IC+4-PBA


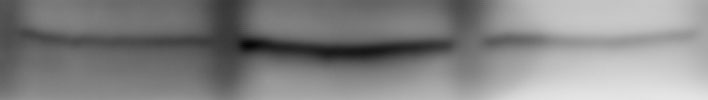
 GRP78


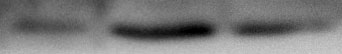
 Beclin1


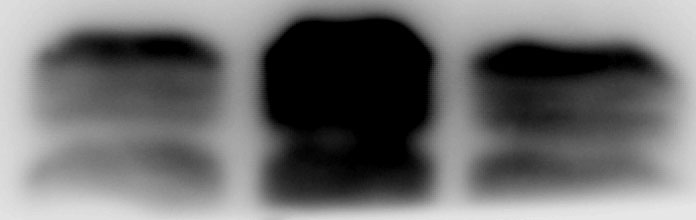
 LC3I/II


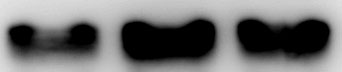
 P62


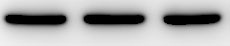
 GAPDH

(5).

N IC IC+4-PBA


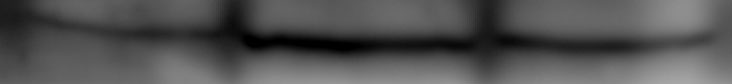
 GRP78


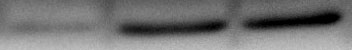
Beclin1


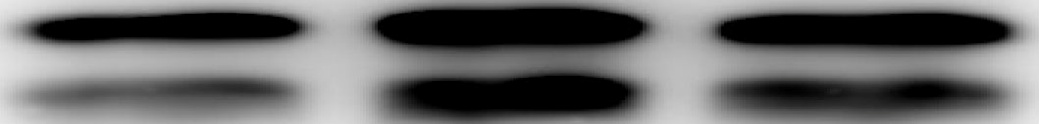
 LC3I/II


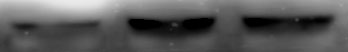
 P62


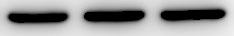
 GAPDH

Fig.3

IL-6

N IC IC+4-PBA


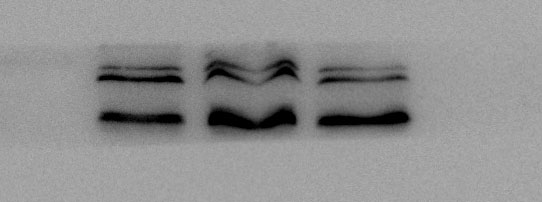


NF-Kb

N IC IC+4-PBA


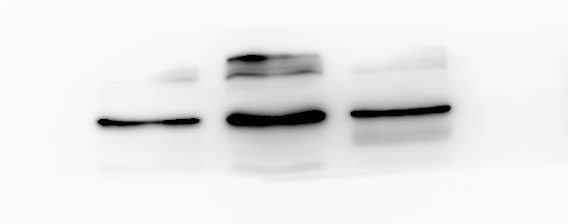


TNFa

N IC IC+4-PBA


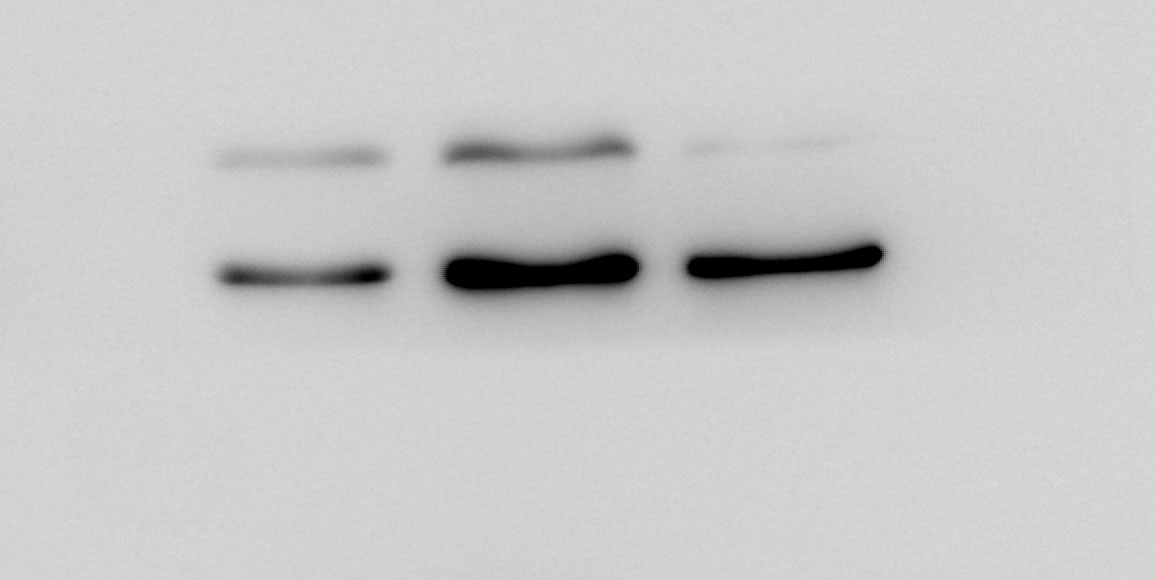


HO-1

N IC IC+4-PBA


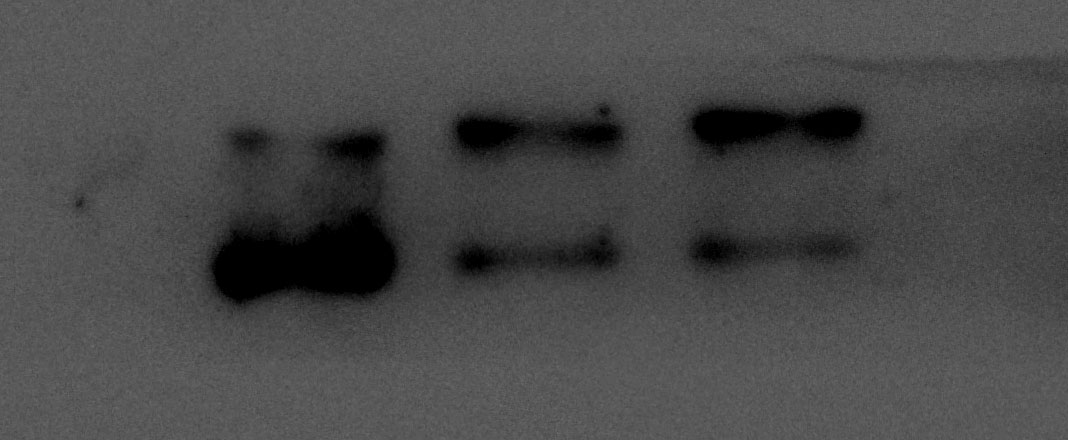


NQO-1

N IC IC+4-PBA


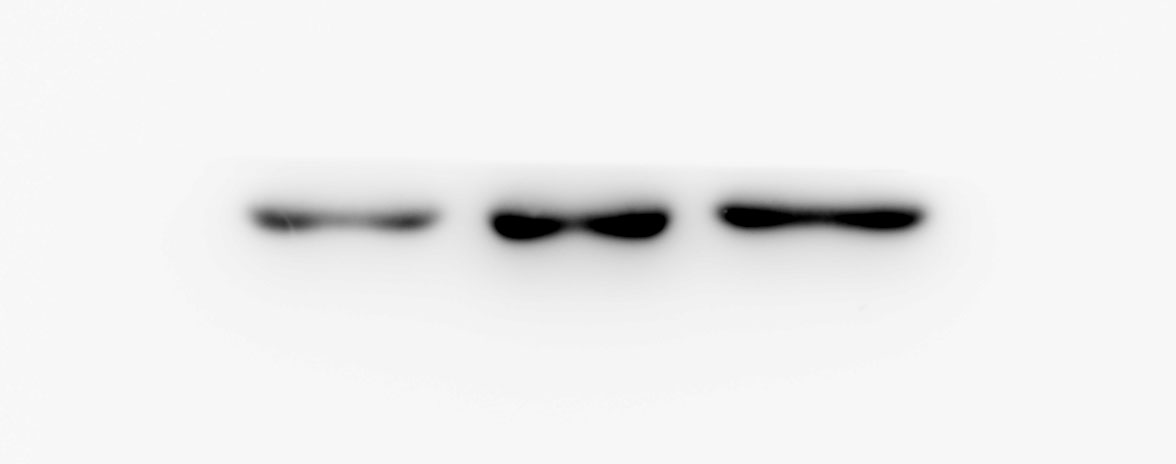


GAPDH

N IC IC+4-PBA


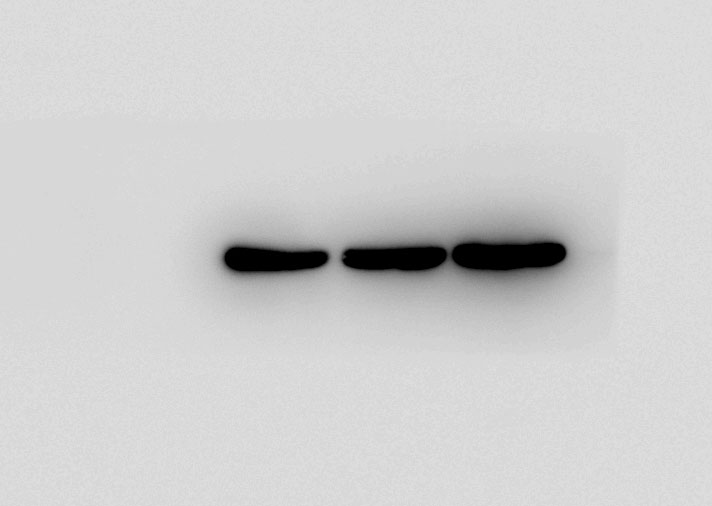


(2).

N IC IC+4-PBA


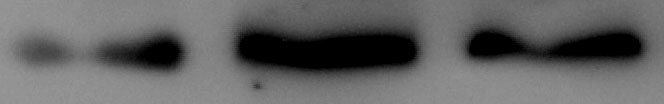
 IL-6


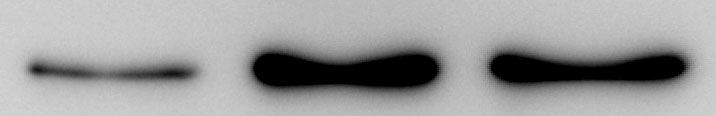
 NF-kB


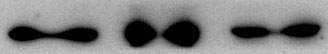
 TNFa


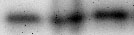
 HO-1


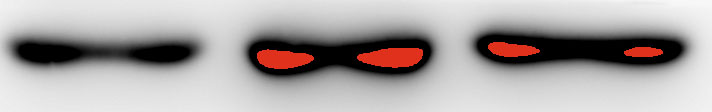
 NQO-1


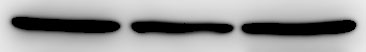
 GAPDH

(3).

N IC IC+4-PBA


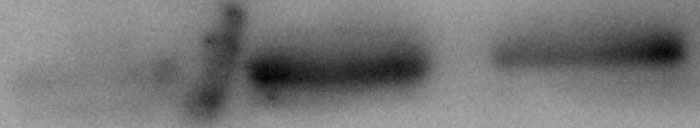
 IL-6


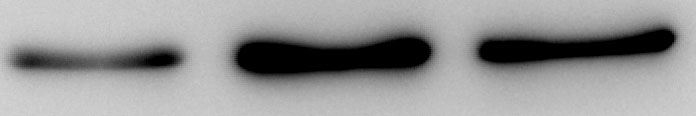
 NF-kB


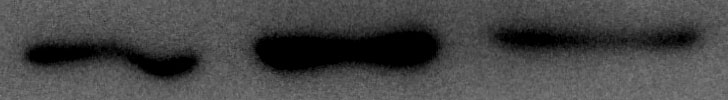
 TNFa


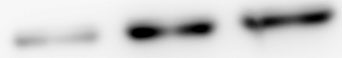
 HO-1


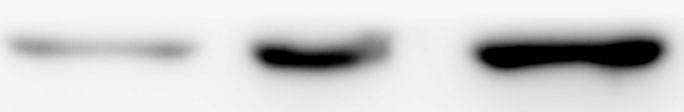
 NQO-1


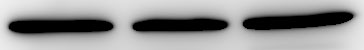
 GAPDH

(4).

N IC IC+4-PBA


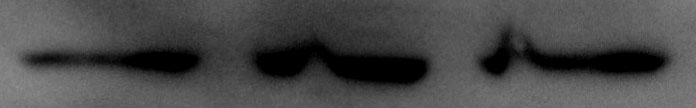
 IL-6


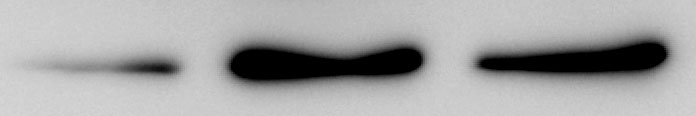
 NF-kB


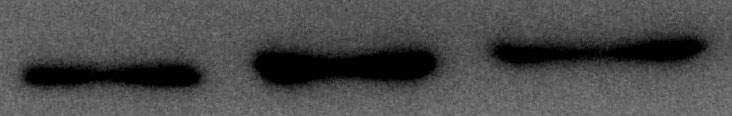
 TNFa


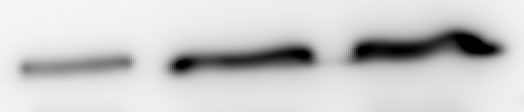
HO-1


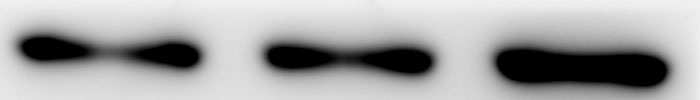
 NQO-1


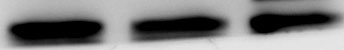
 GAPDH

(5).

N IC IC+4-PBA


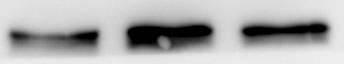
 IL-6


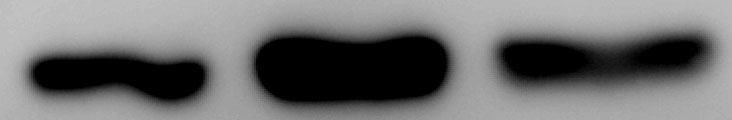
 NF-kB


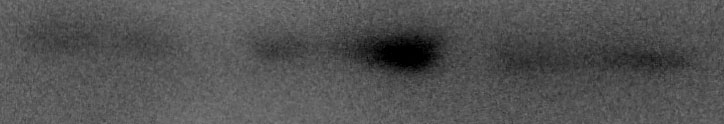
 TNFa


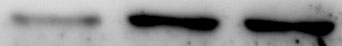
 HO-1


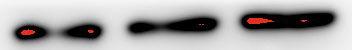
 NQO-1


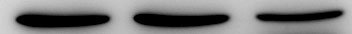
 GAPDH

Fig.4

HE staining

N IC IC+4-PBA


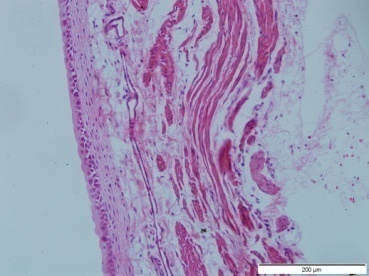

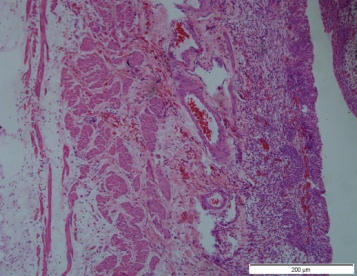

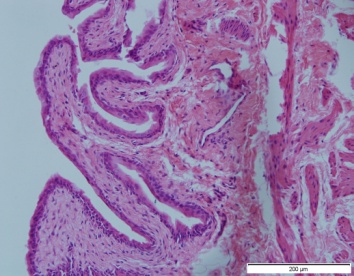


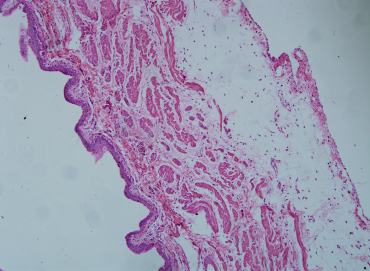

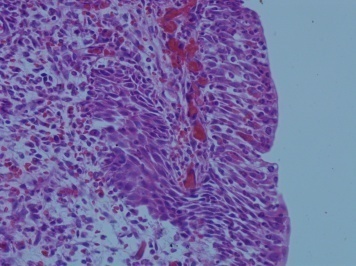

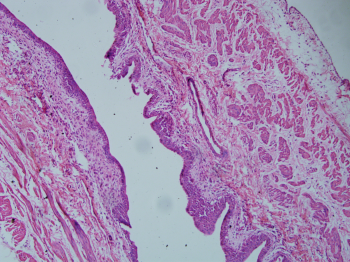


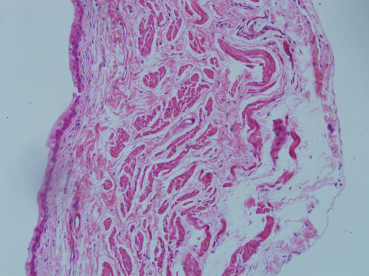

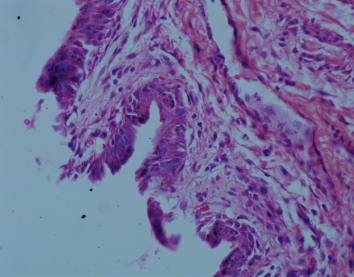

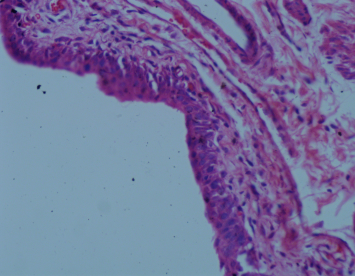


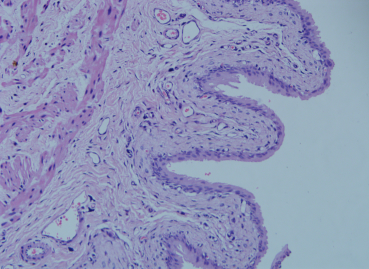

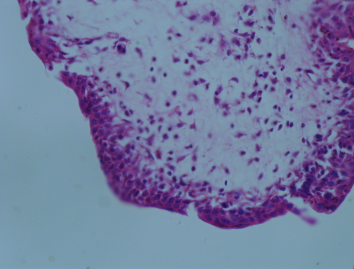

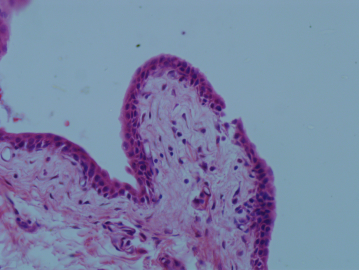


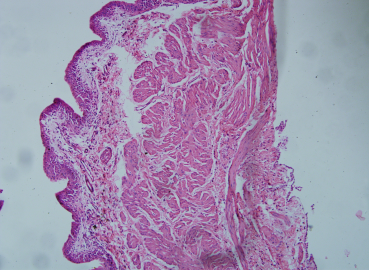

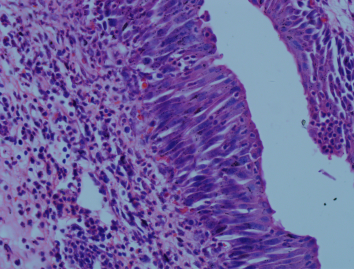

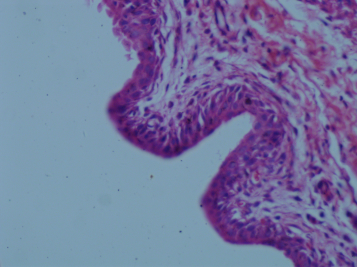


Toluidine blue staining

N IC IC+4-PBA


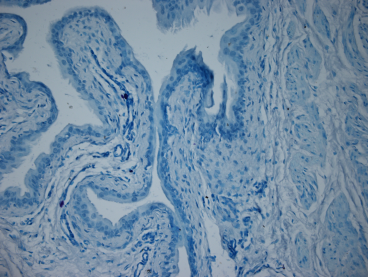

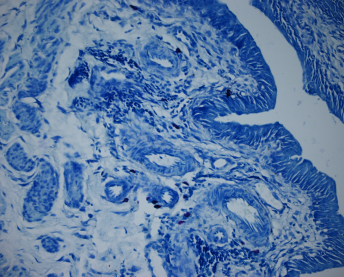

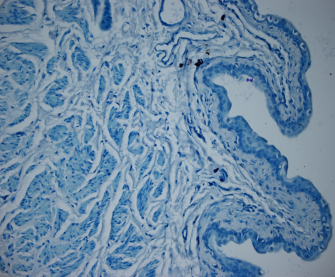


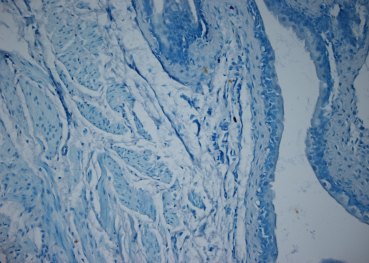

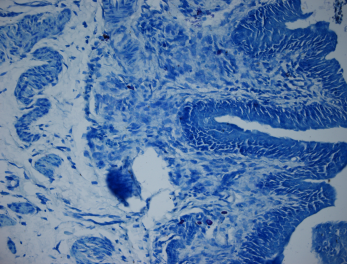

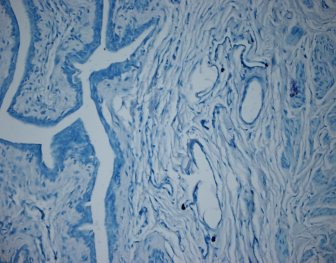


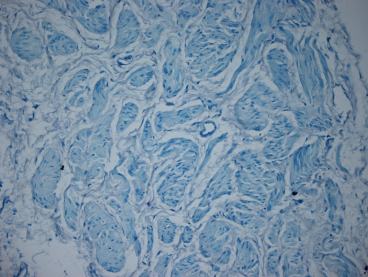

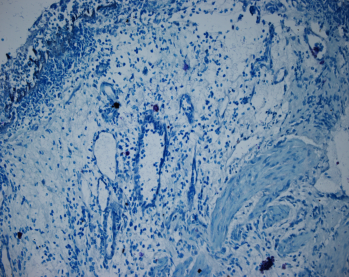

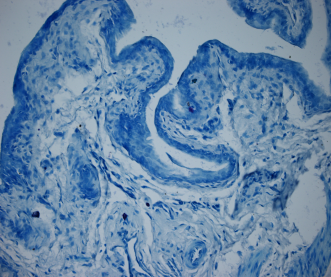


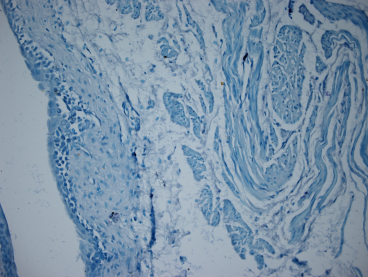

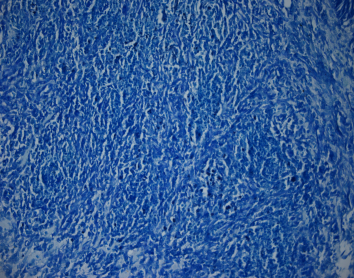

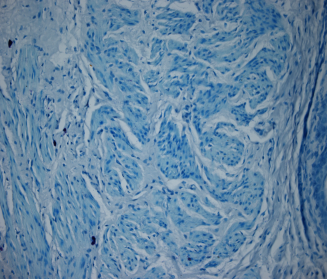


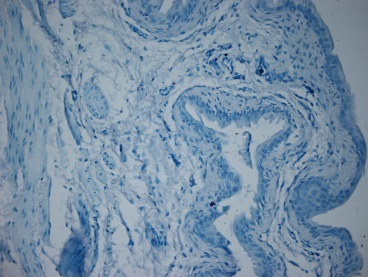

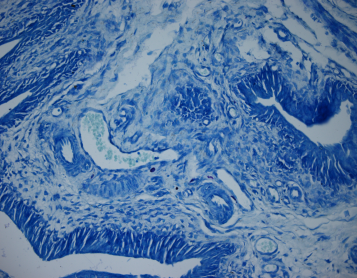

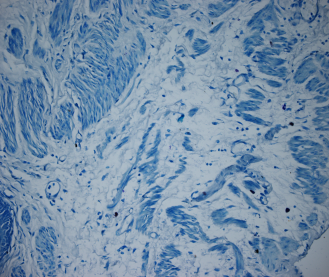


Fig.5

Bax

N IC IC+4-PBA


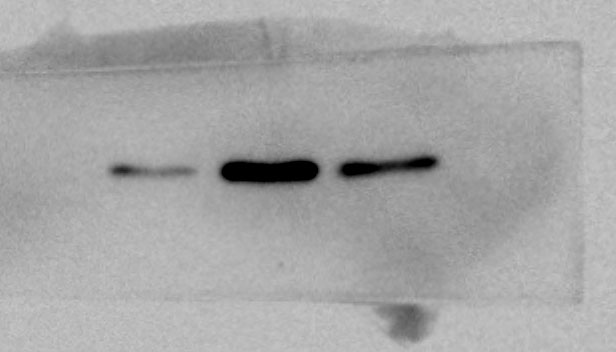


Bcl2

N IC IC+4-PBA


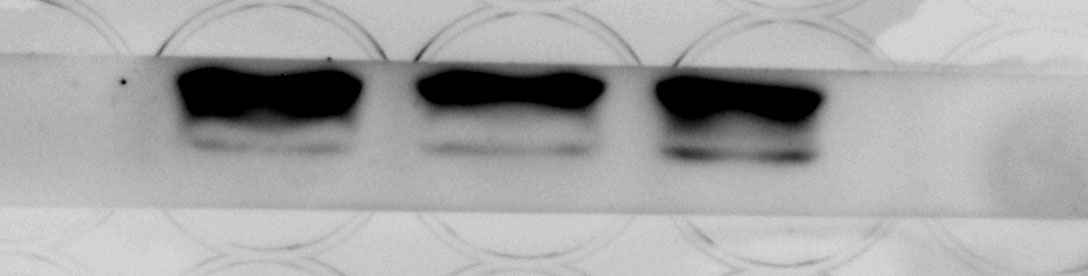


Caspase 3

N IC IC+4-PBA


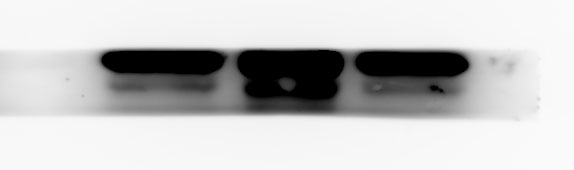


GAPDH

N IC IC+4-PBA


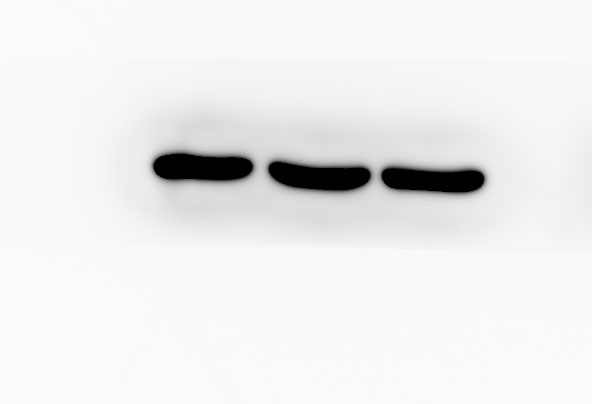


(2).

N IC IC+4-PBA


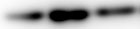
 Bax


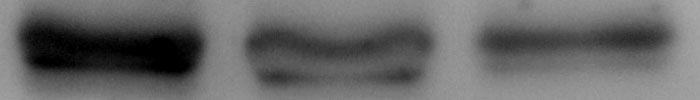
 Bcl2


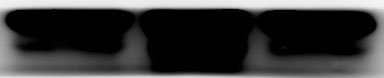
 caspase 3


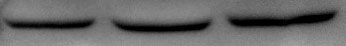
 GAPDH

(3).

N IC IC+4-PBA


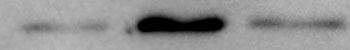
 Bax

Bcl2

caspase 3

GAPDH

(4).

N IC IC+4-PBA

Bax

Bcl2

caspase 3

GAPDH

(5).

N IC IC+4-PBA

Bax

Bcl2

caspase 3

GAPDH

TUNEL staining

N IC IC+4-PBA

Fig.6

(1)

N

IC

IC+4-PBA

(2)

N

IC

IC+4-PBA

(3)

N

IC

IC+4-PBA

(4)

N

IC

IC+4-PBA

(5)

N

IC

IC+4-PBA
